# Dentofacial and skeletal pattern in African descendants from southeastern Brazil: clinical prospective study

**DOI:** 10.1590/2177-6709.26.3.e2119288.oar

**Published:** 2021-06-30

**Authors:** Teresa Cristina Pereira de OLIVEIRA, Flávio de Mendonça COPELLO, Isabela Maria de Carvalho Crusoé SILVA, Lincoln Issamu NOJIMA, Matilde da Cunha Gonçalves NOJIMA

**Affiliations:** 1 Universidade Federal do Rio de Janeiro, Departamento de Odontopediatria e Ortodontia (Rio de Janeiro/RJ, Brazil).; 2 Odontoclínica Central da Marinha (Rio de Janeiro/RJ, Brazil).

**Keywords:** Face, Cephalometry, Brazil, African continental ancestry group

## Abstract

**Objective:**

The aim of this study was to evaluate characteristics of African-Brazilians young adults with excellent dental occlusion, including bimaxillary protrusion; compare them to European-American Caucasian standards, and determine whether there is sexual dimorphism in the display of this phenotype.

**Methods:**

Lateral cephalometric radiographs were obtained from 43 African-Brazilians within military personnel (28 males and 15 females, average age 22.4 ± 3.4 years) with normal occlusion, selected from a group of 394 volunteers. Thirty-one angular and linear measurements were evaluated. Student’s *t*-test for independent samples was used to compare results with those established by European-American standards, previously described in the literature.

**Results:**

Considering the dentoalveolar pattern, seven angular and six linear measurements showed statistically significant differences (*p*< 0.001) when compared to Caucasian cephalometric standards. African-Brazilians’ subjects showed lower cranial base angle (SNAr = 119.87 ± 5.66º) and anterior cranial base length (SN-distance = 68.63 ± 4.50 mm) (*p*< 0.001). The maxilla (SNA = 88.51 ± 3.23º) and the mandible (SNB = 85.06 ± 3.24º) were protruded in relation to the SN line (*p*< 0.001). Sexual dimorphism was significant for L1.NB (degrees) (*p*< 0.01), and interincisal angle (U1.L1) (*p*< 0.05).

**Conclusion:**

African-Brazilian young adults presented differences regarding dental and craniofacial characteristics, when compared to European-American norms. It can be stated that Caucasian cephalometric norms should not be applied to African-Brazilian faces.

## RESUMO

**Objetivo:**

O objetivo do presente estudo foi avaliar características de jovens adultos afro-brasileiros com oclusão excelente, incluindo protrusão bimaxilar, e compará-los com os padrões caucasianos europeu-americanos, para determinar se há dimorfismo sexual na exibição desse fenótipo.

**Métodos:**

Radiografias cefalométricas laterais foram obtidas de 43 afro-brasileiros militares (28 homens e 15 mulheres, idade média de 22,4 ± 3,4 anos) com oclusão normal, selecionados de um grupo de 394 voluntários. Foram avaliadas 31 medidas angulares e lineares. O teste *t* de Student para amostras independentes foi utilizado para comparar os resultados com os estabelecidos pelos padrões caucasianos europeu-americanos, descrito previamente na literatura.

**Resultados:**

Considerando o padrão dentoalveolar, sete medidas angulares e seis lineares apresentaram diferenças estatisticamente significativas (*p*< 0,001) quando comparadas aos padrões cefalométricos caucasianos. Sujeitos afro-brasileiros apresentaram menor ângulo da base do crânio (SNAr = 119,87 ± 5,66º) e comprimento da base craniana anterior (distância SN = 68,63 ± 4,50 mm) (*p*< 0,001). A maxila (SNA = 88,51 ± 3,23º) e a mandíbula (SNB = 85,06 ± 3,24º) estavam protruídas em relação à linha SN (*p*< 0,001). O dimorfismo de sexo foi significativo para L1.NB (graus) (*p*< 0,01) e ângulo interincisal (U1.L1) (*p*< 0,05).

**Conclusão:**

Jovens adultos afro-brasileiros apresentaram diferenças em relação às características dentárias e craniofaciais, quando comparados às normas europeias-americanas. Pode-se afirmar que as normas cefalométricas caucasianas não devem ser aplicadas às faces de indivíduos afro-brasileiros.

## INTRODUCTION

North American and European cephalometric standards are still widely used in orthodontic planning and extraction decision making, despite the ethnic and racial plurality found in contemporary society. However, cephalometric norms cannot be applied to all individuals due to certain racial characteristics and miscegenation, thus making it necessary to establish specific cephalometric patterns for different ethnic groups.^1^ ^,^ ^2^

Several studies have described dentoalveolar variations in Asian,^3^ ^-^ ^5^ Arabic,^6^ ^-^ ^9^ African,^10^ ^-^ ^15^ African-American^16^ ^-^ ^20^ and African-Brazilian^21,22^ populations. Thereby, the cephalometric norms for some ethnic groups should be regarded carefully. For instance, the American black population derives from the miscegenation of different races found in the United States, with those from different parts of Africa.^23^ Similarly, African descendants living today in Southeastern Brazil are very heterogeneous in morphology, because most of them descend from African Bantu slaves who mixed with Mediterranean European colonizers and Native American Indians. The Bantu people in turn, prevail in two vast regions of the African continent: Mid-Eastern Africa, including the Old Portuguese colonies of Angola and Mozambique, as well as the Congo region; and Western Africa ranging from the Southern coast up to the Guinea Golf.^24^

The 2010 census conducted in Brazil revealed that blacks and browns make up the equivalent of 50.7% of the population. However, the scientific literature is scarce in relation to the craniofacial morphology of Brazilian Afro-descendants,^22^ and the few existing investigations describe only growing subjects. The intense demand for orthodontic treatment by young adults raises the need to evaluate cephalometric pattern concerning individual’s profile of this ethnic group, since some characteristics diagnosed by cephalometric radiographs are highly associated with this population, such as bimaxillary protrusion.^21^ ^,^ ^22^

Bimaxillary protrusion can be described by the forward and proclined positioning of maxillary and mandibular incisors over the basal bone. This condition acts as an important motivating factor for orthodontic treatment, due to the negative esthetic impact of protruding lips and profile convexity.^1^ The low prevalence of bimaxillary protrusion in Caucasians with normal occlusion differs highly from what has been reported in the literature for other ethnic and racial groups, such as relevant information about sexual dimorphism in African phenotype.

Based on the rising demand for orthodontic treatment by adults and the lack of studies for this specific Brazilian ethnic group, the aims of this study were: (1) to evaluate in 2D images the dental and craniofacial characteristics of African-Brazilian young adults with excellent dental occlusion, including bimaxillary protrusion, and compare them to European-American Caucasian standards; and (2) determine whether there is sexual dimorphism in the display of this phenotype.

## MATERIAL AND METHODS

This prospective clinical study was approved by the Ethics in Research Committee of the Institute for General Health Studies at the Federal University of Rio de Janeiro (IESC - UFRJ, statement nº. 66/2011). All subjects gave written informed consent and were aware of the procedures adopted in the present research. The required sample size was determined according to the power analysis at α = 0.05 significance level and 80% power (based on a 5.5º standard deviation and a 4.5º minimum clinically detectable U1.NA difference^24^). The sample size needed for the study was at least 39. A total of 43 volunteers (28 male and 15 female) were selected from 394 Brazilian active duty Navy personnel attending the Naval Central Dental Clinic (Rio de Janeiro - Brazil). 

All subjects were born in southeastern Brazil and answered a questionnaire about their ancestors, in which they affirmed to having African ancestry up to the third generation. The average age in the group was 22.4 ± 3.4 years. Females presented an average age of 22.0 ± 4.3 years, and males, of 22.6 ± 3.1 years. All individuals were in good state of general health. The selection criteria included normal occlusion of first molars and canines (except for the presence of third molars); anterior crowding, slight rotations and small gaps up to 2 mm permitted, distributed over the dental arch; 20 to 30% overbite; 1 to 3 mm overjet; absence of crossbites and previous orthodontic or orthognathic treatments.

Orthodontic records including study casts, lateral cephalometric radiographs, facial and intraoral photographs were obtained for each subject. All lateral cephalometric radiographs were taken by the same operator, using the cephalostat (Ortophos Plus DS; Sirona Dental System, Bensheim, Germany) according to standard regulation of exposure time of 0.4s, X-ray tube voltage of 73 kV, and X-ray tube current of 15 mA, maintaining each individual with teeth placed in maximum intercuspation, lips at rest and Frankfort horizontal plane parallel to the ground, as in natural head position. Digitalized images obtained were 18 cm x 24 cm in size, and stored in TIFF format.

**Figure 1:**
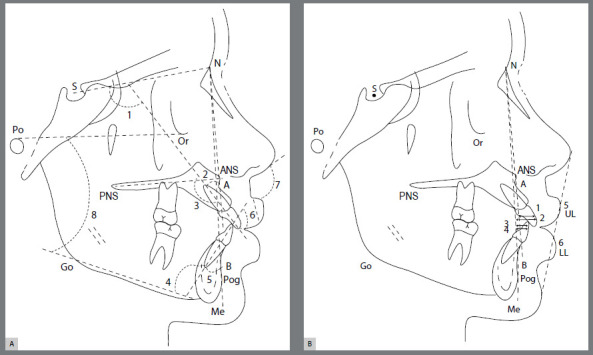
A) Angular measurements used for dental and soft tissue analysis. Reference points: S ( Sella ); N ( Nasion ); Po ( Porion ), Or ( Orbitale ); ANS ( Anterior Nasal Spine ); PNS ( Posterior Nasal Spine ); Pog ( Pogonion ); Go ( Gonion ); Me ( Menton ); A ( Point A Subspinale ); B ( Point B Supramentale ). Reference Lines: SN; NA; NB; Frankfort Horizontal Plane ( Po-Or ); Tweed´s Mandibular Plane ( Go-Me ); Palatal Plane ( ANS-PNS ); Upper incisor (U1); Lower incisor (L1); Ricketts´ Esthetic Plane ( E ). Angular measurements: 1) U1.SN; 2) U1.NA; 3) U1.PP; 4) IMPA; 5) L1.NB; 6) Interincisal Angle (U1.L1); 7) Nasolabial Angle; 8) FMA. B) Linear measurements used for dental and soft tissue analysis. Reference points: S ( Sella ); N ( Nasion ); Po ( Porion ), Or ( Orbitale ); ANS ( Anterior Nasal Spine ); PNS ( Posterior Nasal Spine ); Pog ( Pogonion ); Go ( Gonion ); Me ( Menton ); A ( Point A Subspinale ); B ( Point B Supramentale ). Reference lines: SN; NA; NB; Frankfort Horizontal Plane ( Po-Or ); Tweed´s Mandibular Plane ( Go-Me ); Palatal Plane ( ANS-PNS ); APog Line; Upper incisor (U1); Lower incisor (L1); Ricketts´ Esthetic Plane ( E ). Linear measurements: 1) U1-NA; 2) U1-APog; 3) L1-NB; 4) L1-APog; 5) UL-E; 6) LL-E.

**Figure 2:**
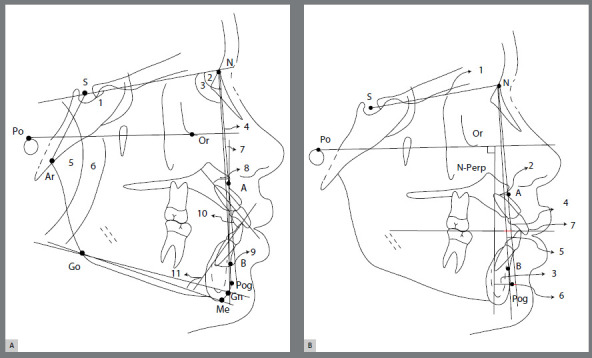
Angular and linear measurements for craniofacial analysis. Reference points: S (Sella); N (Nasion); Ar (Articular), A (Subspinale); B (Supramentale); Go (Gonion); Gn (Gnation); Pog (Pogonion); Or (Orbitale). Reference lines: SN; SAr; NA; NB; APog Line; Frankfort Horizontal Plane (Po-Or); Nperp Line(vertical reference line, i.e., extension of the line perpendicular to the Frankfort Horizontal Plane passing through point N); Occlusal Plane, Upper incisor (U1); Lower incisor (L1); Tweed’s Mandibular Plane (Go-Me); Steiner’s Mandibular Plane (Go-Gn); Long axis of Upper Incisor; Long axis of Lower Incisor. **A**) Angular measurements for craniofacial analysis: 1) SNAr; 2) SNA; 3) SNB; 4) ANB; 5) SNGoGn; 6) FMA; 7) Angle of Convexity (NAPog); 8) U1.NA; 9) L1.NB; 10) Interincisal Angle; 11) IMPA. **B**) Linear measurements for craniofacial analysis: 1) Cranial Base Length SN Distance; 2) A-Nperp Distance; 3) Pog-Nperp Distance; 4) U1-NA Distance; 5) L1-NB Distance; 6) Pog-NB Distance; 7) AO-BO distance (Wits Projection).

All cephalometric tracings were performed digitally by the same operator, using Dolphin Imaging^®^ System 11.0 (Dolphin Imaging, Chatsworth, California, USA). Fifteen radiographs were randomly chosen and measured twice after an interval of two weeks, with the intention to test operator calibration for each value of interest, by means of intraclass coefficient correlation (ICC). Tracings were limited to 5 to 10 per day, to minimize fatigue-induced errors. Dolphin Imaging^®^ System corrected X-ray distortions, so that angular and linear measurements were not altered. Cephalometric landmarks, reference lines and planes, angular and linear variables used in the study are presented in Figures 1 and 2. The nasolabial angle was included to investigate soft tissue convexity and the position of upper and lower lips, according to the esthetic plane proposed by Ricketts.^25^

Data normality of all variables was confirmed using Kolmogorov-Smirnov test. Descriptive analysis was performed, so that the measures of central tendency (mean and standard deviations) represented the most common characteristics found in the studied group. As the sample displayed normal distribution, Student’s *t*-test for independent samples was used to assess differences found in African-Brazilian subjects as opposed to the European-American Caucasians norms defined in the literature,^25^ ^-^ ^29^ and to evaluate sexual dimorphism in the study. Statistical analysis was carried out on SPSS software, version 16.0 (Chicago, Ill). A 5% level of significance was adopted.

## RESULTS

ICC analyses demonstrated excellent rates of reproducibility, with values > 0.9 for all variables.

Considering the dentoalveolar pattern, African-Brazilians presented statistically significant differences compared to Caucasian cephalometric standards (*p*< 0.001). The maxillary and mandibular incisors were significantly more proclined and protruded in African-Brazilians than in European-Americans, as was observed for linear and angular variables in Table 1. Thus, the interincisal angle was more acute (average = 117 ± 7.2º) compared to Steiner´s norm (U1.L1 = 131º). Bimaxillary protrusion was evidenced in African-Brazilians with excellent occlusion (Table 1). Regarding soft tissue profile, the proclination and protrusion of underlying dentoalveolar structures contributed to a significant decrease of the nasolabial angle (89.04 ± 9.33º) and to the projection of upper and lower lips beyond Rickett’s esthetic plane (Table 1).

**Table 1:** Descriptive data analysis and comparisons for angular (degrees) and linear (mm) variables related to dentofacial pattern of African-Brazilians adults, compared to the European-American cephalometric norms defined by Riedel^26^ (1952), Tweed^27^ (1954), Downs^28^(1956), Ricketts^25^ (1960), and Steiner^29^ (1960).

Variable	African-Brazilians (adults) (n=43)	European-American Norms	
	Mean ± SD	Mean	t
U1.SN (degrees)	114.45 ± 5.38	104	12.73***
U1.NA (degrees)	25.92 ± 4.51	22	5.70***
U1.PP (degrees)	115.84 ± 5.31	109	8.44***
IMPA (degrees)	99.31 ± 5.82	90	10.49***
L1.NB (degrees)	32.91 ± 5.56	25	9.32***
U1.L1 (degrees)	117.71 ± 7.2	131	-12.08***
U1-NA (mm)	8.20 ± 1.98	4	13.86***
U1-APog (mm)	9.92 ± 2.50	2.7	18.87***
L1-NB (mm)	9.19 ± 2.41	4	14.09***
L1-APog (mm)	7.15 ± 2.37	1	16.98***
FMA (degrees)	25.58 ± 4.36	25	0.873
Nasolabial angle (degrees)	89.04 ± 9.33	102	-9.09***
UL-E (mm)	1.26 ± 2.71	-7	19.94***
LL-E (mm)	4.23 ± 2.75	-2	14.80***

Values are expressed in degrees or mm, mean ± standard deviation (SD). Student’s *t*-test for independent samples between groups. ***p < 0.001.

African-Brazilians’ subjects showed lower cranial base angle (SNAr = 119.87 ± 5.66º) and anterior cranial base length (SN-distance = 68.63 ± 4.50 mm) (*p*< 0.001). The maxilla (SNA = 88.51 ± 3.23º) and the mandible (SNB = 85.06 ± 3.24º) were protruded in relation to the SN line (*p*< 0.001). Regarding the Ricketts’ Nperp line, the protrusion of the maxilla was less evidenced (A-Nperp = 1.19 ± 2.79 mm) (*p*< 0.01), with no significantly difference for the mandible (Pog-Nperp = -3.33 ± 5.32 mm) when compared to the European-American standard (Table 2).

**Table 2:** Descriptive data analysis and comparisons for angular (degrees) and linear (mm) variables related to craniofacial pattern of African-Brazilians’ subjects, compared to the European-American cephalometric standards.

Variable	African-Brazilians (adults) (n=43)	European-American Norms	
	Mean ± SD	Mean	t
SNAr (degrees)	119.87 ± 5.66	124	-4.7 ***
SN-distance (mm)	68.63 ± 4.50	77.3	-12.62***
SNA (degrees)	88.51 ± 3.23	82	13.18***
A-Nperp (mm)	1.19 ± 2.79	0	2,80**
SNB (degrees)	85.06 ± 3.24	80	10.23***
Pog-Nperp (mm)	-3.33 ± 5.32	-4	0.81NS
Pog-NB (mm)	0.45 ± 1.57	4	-14.73***
ANB (degrees)	3.46 ± 1.70	2	5.62***
Angle of Convexity (degrees)	6.12 ± 4.08	0	9.83***
Wits Projection (mm)	0.10 ± 2.27	-1	3.19**
SN-GoGn (degrees)	26.47 ± 4.72	32	-7,65***
FMA (degrees)	25.58 ± 4.51	25	0.87 NS
U1.NA (degrees)	25.92 ± 4.51	22	5.70***
U1-NA (mm)	8.20 ± 1.98	4	13.86***
L1.NB (degrees)	32.91 ± 5.56	25	9.13***
L1-NB (mm)	9.19 ± 2.41	4	14.09***
IMPA (degrees)	99.31 ± 5.82	90	10.49***
U1.L1 (degrees)	117.71 ± 7.21	131	-12.08***

Values are expressed in degrees or mm, mean ± standard deviation (SD). Student´s *t*-test for independent samples between groups. *p* ≥ 0.05 non-significant (NS). * significant at *p* <0.05; ** significant at *p* <0.01; *** significant at *p* < 0.001.

Sexual dimorphism was significant for L1.NB (degrees) (*p*< 0.01), and interincisal angle (U1.L1) (*p* < 0.05). The larger proclination of mandibular incisors in women (L1.NB = 35.91 ± 6.02º) contributed to an even more acute interincisal angle, giving females a more characteristic aspect of bimaxillary protrusion in relation to males (Table 3). The anterior cranial base was significantly shorter in females (SN-distance = 64.94 ± 2.60 mm) (*p*<0.001). Females showed a greater protrusion of the skeletal bases, when compared to the Nperp line for the maxilla (A-Nperp=2.82±2.38*mm*) (*p*< 0.01) and mandible (Pog-Nperp=-0.64±3.17 mm) (*p*<0.05) (Table 4).

**Table 3:** Descriptive data analysis and comparisons for angular (degrees) and linear (mm) variables related to dentofacial pattern for both genders of African-Brazilian young adults.

Variable	Male (n=28)	Female (n=15)	t	P
	Mean ± SD	Mean ± SD		
U1.SN (degrees)	114.12 ± 5.07	111.04 ± 6.05	-0.529	NS
U1.NA (degrees)	26.06 ± 3.96	25.66 ± 5.54	-0.279	NS
U1.PP (degrees)	115.30 ± 4.57	116.86 ± 6.53	-0.919	NS
IMPA (degrees)	98.16 ± 5.36	101.47 ± 6.21	-1.82	NS
L1.NB (degrees)	31.30 ± 4.66	35.91 ± 6.02	-2.78	**
U1.L1 (degrees)	119.41 ± 6.54	114.52 ± 7.52	2.21	*
U1-NA (mm)	8.25 ± 2.06	8.10 ± 1.88	0.239	NS
U1-APog (mm)	9.81 ± 2.65	10.12 ± 2.28	-0.377	NS
L1-NB (mm)	9.06 ± 2.10	9.42 ± 2.98	-0.460	NS
L1-APog (mm)	7.00 ± 2.28	7.45 ± 2.60	-0.591	NS
FMA (degrees)	25.97 ± 4.61	24.84 ± 3.90	0.811	NS
Nasolabial angle (degrees)	87.40 ± 9.64	92.10 ± 8.17	-1.60	NS
UL-E (mm)	1.13 ± 2.81	1.49 ± 2.60	-0.553	NS
LL-E (mm)	4.06 ± 2.69	4.55 ± 2.94	0.407	NS

Values are expressed in degrees or mm, mean ± standard deviation (SD). Student´s t-test for independent samples between groups. NS, not significant. ** P < 0.01; * P < 0.05.

**Table 4:** Descriptive data analysis and comparisons for angular (degrees) and linear (mm) variables regarding craniofacial pattern for both genders of African-Brazilian young adults.

Variable	Male (n=28)	Female (n=15)	P
	Mean ± SD	Mean ± SD	
SNAr angle (degrees)	119.13 ± 6.25	121.24 ± 4.21	0.252 NS
SN-distance (mm)	70.61 ± 4.04	64.94 ± 2.60	0.000***
SNA (degrees)	88.05 ± 3.36	89.37 ± 2.89	0.207 NS
A-Nperp (mm)	0.32 ± 2.63	2.82 ± 2.38	0.004**
SNB (degrees)	84.84 ± 3.59	85.46 ± 2.53	0.554NS
Pog-Nperp (mm)	-4.78 ± 5.71	-0.64 ± 3.17	0.013*
Pog-NB (mm)	0.63 ± 1.74	0.11 ± 1.18	0.310 NS
ANB (degrees)	3.22 ± 1.52	3.91 ± 7.34	0.208 NS
Angle of Convexity (degrees)	5.47 ± 3.83	7.34 ± 4.39	0.157 NS
Wits Projection (mm)	0.23 ± 2.46	-0.12 ± 1.94	0.625 NS
SN-GoGn (degrees)	26.27 ± 4.86	26.83 ± 4.60	0.719 NS
FMA (degrees)	25.97 ± 4.61	24.84 ± 3.90	0.422 NS
U1.NA (degrees)	26.06 ± 3.96	25.66 ± 5.54	0.781 NS
U1-NA (mm)	8.25 ± 2.06	8.10 ± 1.88	0.812 NS
L1.NB (degrees)	31.30 ± 4.66	35.91 ± 6.02	0.008**
L1-NB (mm)	9.06 ± 2.10	9.42 ± 2.98	0.648 NS
IMPA (degrees)	98.16 ± 5.36	101.47 ± 6.2	0.075 NS
U1.L1 (degrees)	119.41 ± 6.54	114.52 ± 7.52	0.032*

Values are expressed in degrees or mm, mean ± standard deviation (SD). Student´s t-test for independent samples between groups. P ≥0.05 - non-significant (NS). * significant at P <0.05; ** significant at P <0.01; *** significant at P <0.001.

## DISCUSSION

There is vast literature^2-18,20-22^ of scientific articles proposing cephalometric norms for Caucasian and non-Caucasian ethnic groups. To our knowledge, this may be considered the first cephalometric study to include bimaxillary prevalence in young adult African descendants with excellent occlusion in southeastern Brazil. Other research previously established craniofacial cephalometric norms for adolescent African-Brazilians.^21^ ^,^ ^22^

The inclusion criteria chosen in the present study for this group of African-Brazilians selected from active duty military personnel included: black ancestry up to the third generation, lack of previous orthodontic treatment, southeastern origin, and age ranging from 18 to 30 years. The predominance of males (n=28) over females (n=15) reflect the prevalence rate found in the Brazilian Navy, where subjects were selected. Such restrictive inclusion criteria when applied to populations with high miscegenation tend to limit significantly sample sizes. Nevertheless, there are recent literature reports using groups of similar sizes.^13^ ^,^ ^21^ ^,^ ^22^

African-Brazilians with excellent occlusion showed lower cranial base angle (SNAr) and shorter cranial base (SN distance), when compared to the Caucasian standard (Table 2). The results for the cranial base length are in agreement with previous investigations,^4^ ^,^ ^5^ ^,^ ^8^ which also revealed that the melanodermas’ cranial base is significantly smaller in relation to the leucodermas. This feature confers a posterior position to the Nasion point (N), influencing all measures related to the Sela (S) point or the SN line. The lower angulation of the middle cranial fossa, however, contradicts the findings of Enlow^30^ (1982), which associates a high obtuse SNAr angle with the greater forward and downward displacement of the posterior cranial base, making the middle cranial fossa less angulated and flatter, causing the mandibular branch to rotate back and down. This feature could contribute to shift the mandible to a retrognathic position, thus being the predominant pattern in Class I blacks. The present study found lower values ​​for the cranial base deflection angle (SNAr = 119.87 ± 5.66º) for the Brazilian Afro-descendants and it would be associated with the horizontal facial growth pattern and greater mandibular prognathism. These findings indicated the morphological heterogeneity of Brazilian melanoderma and reinforce the need for specific cephalometric norms for ethnic groups from different regions.

Regarding the maxilla and mandible, the African-Brazilians presented maxillary and mandibular bases significantly more protruded than the Caucasian standard (Table 2). Similar results were found in previous studies that evaluated other melanoderma populations.^12^ ^,^ ^13^ ^,^ ^15^ High values ​​for SNA and SNB are expected when the cranial base is significantly decreased.^5,6^ The maxillary protrusion was confirmed with the A-Nperp distance, however, in a less expressive way, when it was compared to the SNA angle. On the other hand, the mandibular prognathism was not significant when considered Pog-Nperp variable.

The present results showed highly significant differences between African-Brazilians and the European-American standards on all variables (linear and angular) (*p*< 0.001) related to the dentoalveolar pattern (Table 1). When compared to Caucasians, African-Brazilians have significantly more proclined and protruded maxillary and mandibular incisors over the respective basal bone, and consequently more acute interincisal angles. These results support the findings of previous investigations^12^ ^,^ ^13^ ^,^ ^15^ ^,^ ^17^ ^,^ ^18^ ^,^ ^22^ on African-descent populations, which found that bimaxillary protrusion resulted from more labial positioning of maxillary and mandibular incisors. However, other studies found excessive protrusion and proclination only of lower incisors.^10^ ^,^ ^20^

Sexual dimorphism was not observed in most of the analyzed variables, except for angular measurements L1.NB and U1.L1, which support the findings of previous studies^9^ ^,^ ^11^ ^,^ ^13^ that established the high proclination of lower incisors as the main contributor to more acute interincisal angles in females, including in other ethnic groups (Table 3). This difference was statistically significant between genders in the present study and suggests that, among African-Brazilians, women have a greater tendency to present more pronounced bimaxillary protrusion than men.

Soft tissue profile analysis revealed that Brazilians of African descent have a significantly diminished nasolabial angle, and a larger bilabial projection into Ricketts’ Esthetic Plane when compared to European-American standards (Table 1). This confirms the larger projection of soft tissue as a result of dentoalveolar protrusion found on southeastern African-Brazilians with excellent occlusion, as opposed to African populations previously described in the literature.^12^ ^,^ ^17^ ^,^ ^18^ ^,^ ^20^ ^,^ ^22^ These characteristics provided a bilabial protrusion and subsequent soft tissue profile convexity to the group considered in the present research.

In the present study, there was no statistical difference (*p*> 0.001) comparing African-Brazilians and Caucasians, with regard to divergence of the Frankfort Mandibular Plane (FMA). The mean value found for this sample (25.68 ± 4.36º) is within the norm proposed by Tweed (FMA = 25^o^), which indicates that the pronounced incisor projection is not related to discrepancies in anterior facial height or clockwise mandibular rotations. It can be suggested the association to the more anterior positioning of the maxilla with respect to the cranial base. There are some differences between the vertical growth pattern presented in this study, and what was found in the literature regarding African or African-American groups. Many studies found more obtuse values for Frankfort Mandibular Plane angle (FMA) in African^11^ ^,^ ^12^ ^,^ ^15^ and African-American^16^ ^,^ ^17^ subjects when compared to Caucasians. However, there are records of hypodivergence, horizontal facial growth pattern and low values of Frankfort Mandibular Plane angle (FMA) in African^13^ and African-Brazilian^22^ populations. This emphasizes the importance of determining specific cephalometric standards for each ethnic group according to its geographic origin.

The limitation found in this study was the possible magnification difference of the devices used to obtain the radiographic images between this research and the studies used as European standard. However, these same standard studies are used in the orthodontist’s routine. Besides that, it would be interesting to compare this African-Brazilian group to another African standards and with a control group of white southeastern Brazilians.

## CONCLUSIONS

African-Brazilian young adults presented differences regarding dental and craniofacial characteristics when compared to European-American norms.

European-American cephalometric norms do not apply to these individuals and therefore should not be used as references to orthodontic treatment planning for this specific ethnic group, in which a bimaxillary protrusion is more acceptable than for the Caucasian population.

African-Brazilian women revealed larger lower incisor proclination and smaller interincisal angles compared to men. 
